# Male Equivalent Polycystic Ovarian Syndrome: Hormonal, Metabolic and Clinical Aspects

**DOI:** 10.22074/ijfs.2020.6092

**Published:** 2020-07-15

**Authors:** Federica Di Guardo, Lilliana Ciotta, Morena Monteleone, Marco Palumbo

**Affiliations:** Department of General Surgery and Medical Surgical Specialties, University of Catania, Catania, Italy

**Keywords:** Androgenetic Alopecia, Insulin Resistance, Polycystic Ovarian Syndrome

## Abstract

Recent studies identified the presence of a male polycystic ovarian syndrome (PCOS), which mainly affects men whose female relatives are afflicted with PCOS, caused by genes responsible for the susceptibility of this syndrome in women. Similar hormonal, metabolic, and clinical alterations occurring in PCOS women have also been reported in their male relatives, suggesting a association between the male and female forms of the syndrome. Although the remarkable clinical manifestation of the male equivalent PCOS is diagnosed by the early-onset androgenetic alopecia, characterized by hair recession, pronounced hypertrichosis, insulin resistance, biochemical and hormonal abnormalities, the hormonal/metabolic profile is still controversial. Men affected by early-onset androgenetic alopecia (AGA) are at risk of developing hyperinsulinemia, insulin-resistance, dyslipidaemia, and cardiovascular diseases. However, there is no consensus on the association of male equivalent PCOS with hypertension and obesity. Moreover, reduced levels of sex hormone-binding globulin have been detected in these male patients, accompanied by increased free androgens. Conversely, literature reported lower concentrations of testosterone in male equivalent PCOS when compared with the normal range, indicating a crucial role for the conversion of cortical androgens. Finally, further studies are warranted to investigate a possible link among AGA, metabolic/hormonal alterations, and acne. Our study assessed the hormo- nal, metabolic and clinical aspects of male equivalent PCOS syndrome reported in the literature to evaluate similar and divergent elements involved in the female version of the syndrome.

## Introduction

Polycystic ovarian syndrome (PCOS) is a well-known endocrine disorder characterized by the changes in menstrual cycle, altered ultrasound of the ovaries, and clinical and/or biochemical abnormalities resulting from hyper-androgenism (1). Although the alteration of gonadotropin levels, in terms of luteinizing hormone (LH) elevation respect follicle stimulating hormone with respect to follicle stimulating hormone (FSH), seems to be the most representative hormonal sign, PCOS develops in a more complex metabolic background determined by obesity and insulin resistance. These last two elements play a fundamental role in disease pathogenesis, leading to the development of the long-term complications, such as cardiovascular diseases (CVDs) and type II diabetes mellitus (DM II) (2). The scientific scenario has been recently focused on the involvement of a genetic component in the etiology of PCOS, identifying the existence of a male equivalent PCOS resulting from the heredity of susceptibility genes responsible for the pathogenesis of the disease in male relatives of women with PCOS (3). Although similar clinical characteristics of PCOS observed in women have been found in male subjects affected by male equivalent syndrome, the precise mechanism of the hormonal and metabolic backgrounds in these patients has not been yet established (4). The current study aimed to highlight the hormonal, metabolic and clinical aspects of male equivalent PCOS, trying to point out similar and divergent elements from the female syndrome.

## Data sources

The presented study represents a review about male equivalent PCOS. We searched research articles published in MEDLINE (PubMed), EMBASE, IBECS, BIOSIS, Web of Science, SCOPUS, and Grey literature (Google Scholar; British Library) from 2000 to May 2019. We used the terms ‘‘PCOS’’, ‘‘PCOS male equivalent syndrome’’, and “Androgenic alopecia” as appropriate medical subject headings or equivalent subject heading/ thesaurus terms. These terms mentioned earlier were combined with ‘male’, ‘metabolic alterations’, ‘endocrine’, ‘symptoms’ and ‘signs’. The reference list of the available primary studies was reviewed to eventually find the additional relevant citations. We considered the following outcomes: hormonal pattern, metabolic profile, disease symptoms, and signs.

## Screening of abstracts for eligibility

Original papers, meta-analyses, and published reviews were considered. In case of duplicate publications produced by the same team, the latter study was included. Case reports were not considered. Two authors (F.D.G and L.C.) independently extracted the data from the remaining studies. Disagreements about the inclusion or exclusion of articles were solved by consensus, and in the case of, a third reviewer (M.P.) was involved.

Finally, all the authors were involved in decisional process for including the relevant studies and a decision was made in the case of all authors’ approval.

## Study selection and eligibility criteria

A set of explicit criteria were used for the selection of the literature: (1) original articles, (2) meta-analysis conducted on human population, (3) adult males at the age range of 18 to 80 years old, and (4) being written in English language.

The investigation identified a total of 48 papers, of which 35 were potentially relevant following an initial evaluation. These met the inclusion criteria and were analyzed. The included studies were divided into three different issues: “Hormonal pattern”, “Metabolic pattern”, and “Clinical signs”.

## Hormonal pattern

Several studies identified the association of male equivalent PCOS with the hormonal markers of insulin resistance and metabolic syndrome. Although the hormonal pattern of female and male individuals is too challenging to compare, the literature reported a similar hormonal background to PCOS in men affected by the early-onset androgenetic alopecia (AGA). This event has been considered a sign of male equivalent PCOS, since it was found that its characteristic (androgenetic alopecia before 35 years of age) is mostly present in men belonging to families where female members have been affected by PCOS, supporting the idea of a genetic transmission (5). A recent case-control study analyzing the hormonal pattern of 57 young men (19–30 years) with early onset AGA, found hormonal parameters similar to the ones of female PCOS, when compared to the agedmatched controls. Besides, as far as the gonadotropin levels are concerned, the results showed a significant increase in LH, accompanied by reduced FSH. With regards to prolactin (PRL) and dehydroepiandrosterone sulfate (DHEAS), higher values were reported, while FSH and sex hormone-binding globulin (SHBG) were lower than in controls (6). In addition, similar to what happens in women with PCOS, an increased function of adrenal glands has been supposed to occur in men with earlyonset AGA considering the augmented presence of two markers of adrenal activity (17αOH-P and DHEAS) found in these subjects (7) (Table 1). Considering the recent literature about hormonal assessment of earlyonset AGA, these studies showed results in accordance with the pattern discussed above. The first study conducted by Starka et al. (8) focused on the levels of SHBG, FSH, testosterone, and epitestosterone in men, showing a higher percentage of men affected by earlyonset AGA with the lower concentrations of the abovementioned values compared with the normal range. In the same way, in a successive study, they found lower levels of FSH, as well as lower concentrations of serum SHBG and higher levels of free androgen index (FAI) (Table 1).

**Table 1:** Hormonal/metabolic pattern of early onset AGA


Studies	Hormonal and metabolic alterations in men with early onset AGA

Sanke et al.(6)	Increased levels of LH, PRL, and DHEAS Reduced levels of FSH and SHBG
Stárka et al.(7)	Increased levels of 17αOH-P and DHEASReduced levels of SHBG
Starka et al.(8)	Low levels of SHBG with consequently elevation of the free androgen indexInsulin resistance
Arias-Santiago et al.(9), Golden et al.(10)	Lower levels of SHBG and metabolic imbalance
Hirsso et al.(11), Matilainen et al.(12), Su et al.(13)	Higher BMI and risk of developing hyperinsulinemia


Hormonal/metabolic pattern the early-onset AGA considering the results of the most significant literature studies. LH; Luteinizing hormone, PRL; Prolactin, DHEAS; Dehydroepiandrosterone sulfate, FSH; Follicle stimulating hormone, SHBG; Sex hormone binding globulin, 17αOH-P ; 17α hydroxyprogesterone, BMI; Body mass index, and AGA; Androgenetic alopecia.

## Metabolic pattern

Higher frequency of reduced insulin sensitivity was reported in men affected by the early-onset AGA associated with the low level of SHBG, introducing the concept of insulin unbalance in these patients (8). Although the relationship between lower SHBG levels and metabolic alterations has not been yet established (14), some studies reported the combination of lower level of SHBG and metabolic unbalance in the earlyonset AGA men, in virtue of the fact that this protein should be involved in mediation of the signaling pathways responsible for glycaemia maintenance (9, 10) (Table 1). According to this, lower SHBG levels may be regarded as a risk factor for the development of hyperglycaemia, insulinresistance, and/or DM II in young patients with AGA. However, among metabolic abnormalities, a higher prevalence of hyperinsulinemia, hypertriglyceridemia, and hypertension has been reported in siblings with PCOS; more specifically they seem to occur frequently in brothers of women with PCOS (11, 15, 16).

In accordance with the last concept, an increased prevalence of metabolic disorders, such as insulin resistance (IR) and obesity, as well as, the early-onset AGA has been reported in first degree of male and female relatives of women with PCOS. Moreover, scientific evidence reported that men with the early-onset AGA and higher body mass index (BMI) have higher risk of developing hyperinsulinemia (11-13). Finally, in contrast with the expectations, the association among higher BMI values, insulin resistance, and higher blood pressure is still controversial (17). However, a study investigating the risk of developing cardiovascular disease (CVDs) among men with the early-onset AGA showed a higher incidence of metabolic syndrome and carotid atherosclerotic plaques, suggesting the importance of the routinely use of color Doppler ultrasound to scan the plaques in these patients (18). Considering all these elements, patients with the early-onset AGA should be screened for metabolic parameters in order to eventually adopt pharmacological and nutritional therapeutics strategies to reduce the risk of developing DM II and CVDs (2, 18, 19).

## Clinical signs

According to the genetic hypotheses on the development of male equivalent PCOS as a result of the heredity of susceptibility genes in women, Duskovà and colleagues described the syndrome in men for the first time. They characterized the clinical characteristic of male equivalent PCOS manifested by the early-onset AGA and identified several featured elements, such as hypertrichosis, IR, biochemical, and hormonal abnormalities (4, 20, 21) (Table 2). However other authors had previously hypothesized its existence (20-22); for example, Lunde et al. reported the occurrence of the early-onset androgenetic alopecia characterized by the hair recession of the frontotemporal region before 35 years of age. This condition is defined by a grade of alopecia higher than III according to the Hamilton–Norwood scale (23, 24) in male members of families in which a considerable number of women had PCOS (5). AGA is determined by a progressive reduction in size of the hair follicles until the vellus transformation of the terminal hair. This event is the consequence of the alteration in hair cycle dynamics, in particular the telogen phase, increasing with a subsequent decrease in the anagen phase duration. Considering that the duration of anagen is responsible for the hair length, the new anagen hair becomes shorter, eventually leading to baldness (5, 24). As discussed in the previous section, the suspicion that the early-onset AGA may represent a clinical marker of IR founded on the results of a case-control study reporting an increased prevalence of hyperinsulinemia and insulinresistance- associated disorders such as dyslipidaemia, hypertension and obesity, in men with the-early onset of alopecia (<35), compared with age-matched controls (8). Moreover, several studies revealed that the condition of IR is also associated with acne in young males and postadolescent men (25, 26).

**Table 2:** Clinical elements of Early onset AGA


Studies	Hormonal and metabolic alterations in men with early onset AGA

Carey et al.(20), Legro et al.(21), Dusková et al.(4)	Hypertrichosis, insulin resistance biochemical, and hormonal abnormalities hyperglycemia/T2DM, and IR
Arias-Santiago et al.(31), Arias-Santiago et al.(18)	Increased risk of CVD due to the metabolic abnormalities increased risk of atheromatosis
Arias-Santiago et al.(31)	Higher levels of aldosterone contributing to the development of hypertension and increased prevalence of hypertension, lipid-lowering medication use, and obesity.


Symptoms and Signs of the early-onset AGA considering the results of the most significant literature studies. IR; Insuline resistence, T2DM; Type II diabetes mellitus, CVDs; Cardiovascular disease .

Additionally, a study by Del Prete et al. (25) found a significant increase in plasma insulin levels, higher BMI, and waist circumference in young men with acne compared to healthy control subjects. Although the investigation of the androgenic profiles in males with acne was normal, this finding let us suppose an independent role of IR in the pathogenesis of the acne in the absence of hyperandrogenism. This mechanism may explain the presence of acne in men affected by the early-onset AGA either in the presence or absence of hyperandrogenism. With regard to the metabolic abnormalities in men with the early-onset AGA, it seems there is high prevalence of metabolic syndrome reflecting the phenotypical pattern of PCOS women (6, 27). According to this result, a metaanalysis reported an increased risk of metabolic syndrome development about 2.3 folds in AGA patients compared to healthy controls (28). However, other studies did not confirm this relation that is strongly well documented in the case of female pattern alopecia (29, 30). What is certainly known is the association of the early-onset AGA with hyperglycemia/ DM II and low levels of SHBG (31) (Table 2).

## Discussion

Considering the evidence mentioned above, male equivalent PCOS may be considered a well-defined entity involving specific hormonal and metabolic patterns, as well as, the signs and symptoms. Although the syndrome was taken into account by more than ten years (21), it has been changeling to recognize its typical elements in order to the fact that the androgens-related alopecia have been considered normal in the male phenotype. Moreover, it is not common that the male category recurs to medical consultation for signs involved in the virilization process (12). In this case, symptoms of PCOS, such as acne, defluvium, and hypertricosis/hirsutism do not affect men as much as women. This may be explained by the fact that in women these symptoms are accompanied by the irregular cycles which, in most of the cases catch the women attention, letting them decide to consult a gynecologist (32). According to the lines above, this syndrome has to be considered a possible pathology in the case of men with PCOS-positive family history and hyper-androgenism signs accompanied by the metabolic alterations and hormonal PCOS pattern.

Although the association of the early-onset AGA and acne with male equivalent PCOS is well documented, less clear data are available for hypertricosis that has to be investigated in detail. On the other hand, in opposed to what we expect, hypertestosteronemia does not represent one of the characteristic element of male equivalent PCOS. According to this, scientific literature reported testosterone values lower than the normal range and higher FAI in men with the early-onset AGA (8). This should be explained by the conversion of cortical androgens, such as DHEAS, which is increased in these subjects, and converted into other compounds and stronger androgens responsible for syndrome sings. Moreover, the difference in testosterone values between men and women affected by the two syndromes is due to the different action mediated by insulin on Theca and Leydig cells, stimulating and inhibiting the steroidogenesis, respectively (33, 34).

What is certain is that men with clinical aspect of the early-onset AGA are at risk of developing CVDs, metabolic syndrome and carotid atherosclerotic plaques (18). Similarly, first degree relatives of women with PCOS report an increased prevalence of insulin resistance, obesity, and early-onset AGA. Moreover, although the association with male equivalent PCOS and hyperinsulinemia is still controversial, several studies demonstrated that men with the early-onset AGA and higher BMI had higher risk of developing insulin resistance (11-13).

In contrast to the expectations, the presented paper highlights that high BMI, obesity, and pathologicrelated symptoms, such as hypertension and high cholesterol levels, do not seem to be typical elements of male equivalent PCOS. In particular, the literature shows only one study conducted on men with the earlyonset AGA, reporting a positive correlation between the levels of diastolic blood pressure and insulin resistance, excluding the BMI parameter. Accordingly, it has been identified a correlation among the levels of diastolic blood pressure, total cholesterol, insuline resistance, and fasting insulinresistance index (FIRI). All these parameters were higher in patients with the early-onset AGA than in controls (35). In addition, a case controlstudy conducted on 80 men with the early-onset AGA reported higher values of diastolic blood pressure and a frequent family history of AGA in non-obese cases than BMI-matched counterparts (36). According to this, we may affirm that high BMI and obesity cannot be defined as typical elements of male equivalent PCOS but when presented, they may contribute to the development of the hormonal, metabolic, and clinical scenario. Finally, an important mention may be reserved to the fertility potential of men affected by AGA; the literature shows that men with moderate to severe AGA have poorer semen quality than patients affected by moderate to mild AGA (36). In this context, it would be interesting to evaluate the potential fertility of men affected by male equivalent PCOS according to typical metabolic and hormonal alterations mentioned earlier.

## Conclusion

Male equivalent PCOS may be defined as a disorder that occurs in male members of a family with a PCOS history, characterized by the clinical signs of androgenism, complete hair loss, and the same hormonal pattern seen in PCOS, except for testosterone levels that seems to be in the subnormal range. The metabolic pattern should be represented by hyperinsulinemia and insulin resistance with a side role for overweight and obesity in the case of occurrence. However, these patients have high risk of developing CVDs, metabolic syndrome, and carotid atherosclerotic plaques. According to this, the early diagnosis of the disease would be necessary to permit the patients to adopt healthy lifestyle preventing the risk of metabolic and cardiovascular events.
